# Muscular Poisons

**Published:** 1861-01

**Authors:** M. Claude Bernard


### ON MUSCULAR POISONS.

BY M. CLAUDE BERNARD.

There exists other poisons which equally destroy the vital properties of the locomotive apparatus, although a totally different histological element becomes the seat of disorganization. We allude to the toxic agents which abolish contractility in the muscular tissue; you will find them infinitely more numerous than those which confine their action to the nerves. Among this class of bodies, digitalis and upas antiar* have already been mentioned; and the attention of American physiologists has lately been drawn by Drs. Hammond and Mitchell, of Philadelphia, to the properties of two other substances which are known by the Indian names of Corroval and Vao. Their action is similar to that of upas, although of greater intensity. The active principle of the veratrum album or veratrine, a substance now frequently employed in practice, also exerts its influence upon the muscular fibre, to the exclusion of all other tissues; and a large number of poisons, with the chemical composition of which we are imperfectly acquainted, evidently belong to the same class.

The small arrows now placed before you were forwarded to us by M. Boussingault. These weapons are extensively used in South America. The nature of the substance with which they are impregnated is still a mystery to us; they are supposed to have been charged with woorara, but their action is of a totally different kind; the muscular, not the nervous, fibres are affected by the wounds which they inflict. Although unable to state in a positive manner what this poison really is, I am tempted to believe that the venom of toads is the substance employed. The Indians of New Grenada are know to

* There exists another kind of upas, which is merely a preparation of strychnia.

make use of a small variety of this animal, which is found in great abundance in that country, for the purpose of poisoning their weapons. After collecting a large number of toads, they impale them upon wooden spits and roast them alive. The heat causes the animal to eject its venom, in which the arrows are dipped. This latter substance is known in fact to exert a most powerful action on the muscular tissue. But the unknown principle with which these arrows are impregnated differs so completely in its properties from those usually attributed to animal poisons, that we shall seize the present opportunity to enter into some considerations on the subject of venoms in general, and that of the toad in particular, our conclusions being the result of the researches we have lately undertaken on this interesting and hithorto obscure part of toxicology.

The effect produced on animals by these poisoned arrows would appear at first sight to be strictly similar to those produced in our own climate by the venom of toads; but when we enquire into the chemical composition of this new substance, we find it to be a perfectly stable compound, the properties of which are not destroyed by plunging it into boiling water or dissolving it in alcohol. The arrows which have been allowed to rest for a certain space of time in this latter solvent, are found to have lost their destructive power, although no change has taken place in their dark-brown color; and the liquid in which they have been kept being gradually evaporated, the solid residue left behind appears to contain the active principle which imbibes them, for on redissolving it in water, a poison is obtained which produces on animals the same effects as the arrows themselves.

But the opinions usually entertained with respect to venoms are entirely opposed to such a result. Animal poisons are in general considered as resembling ferments in their chemical properties, being easily destroyed by the action of heat, and modified by dissolution in alcohol and other powerful solvents. We ought, therefore, in accordance with generally received opinions, to have surrendered our former hypothesis; but however satisfactory the inductions drawn from previously ascertained facts may appear, an experimental verification is always indispensable in sound physiology, even in those cases in which it seems useful and almost absurd to require experimental proofs. Acting in conformity with this rule, which has always directed us up to the present time in our labors, we have examined in its turn the venom of toads for the purpose of ascertaining whether its characteristics were really such as had been supposed, and, contrary to our expectations, we have found it to be soluble in alcohol, and in all other respects as stable a compound as the active principle of these American arrows, for the action of boiling water destroys none of its properties.

It had been supposed, however, that no venom was fatal to the animal itself which secreted it; and as the poisonous arrows produce marked effects upon the toad, we found it necessary to try upon this animal the results produced by the inoculation of its own venom, in order to ascertain whether the opinion generally entertained on this point was scientifically true. The experiments undertaken for this purpose do not allow the slightest doubt to remain on this point. The toad is killed, like other animals, by the inoculation of its own venom; it is, however, true that the action of this poison is infinitely less intense in toads than in frogs, of all other animals those which resemble them most in organization; and it might hence be inferred that a certain degree of truth exists in the above-mentioned opinions. Let us bear in mind, however, that the resistance of the toad to all kinds of poison is infinitely superior to that of the frog; that strychnia, upas, and various other substances act in much smaller doses upon the latter than upon the former animal—a fact which agrees with the lower excitability of the nervous system in toads.

All these phenomena may therefore be reduced to a general principle, which we have strenuously endeavored to inculcate in the course of the present lectures, namely, that no essential differences exist between the homologous tissues of different animals; that properties observed in one case are found to exist throughout the entire scale of being, although, considerable variations in their intensity are met with even in the case of neighboring species. You also perceive that our knowledge of venoms is less advanced than the other branches of toxicology, and that is urgent to repeat a new series of experiments on each of those substances in particular; for we are not prepared, up to the present moment, to attribute the properties enjoyed by the venom of toads to the whole series of animal poisons; further researches are indispensable to settle the question.

Let us now, gentlemen, after this digression, resume the explanation of the effects produced by muscular poisons. The principal result of their action is sudden arrest of the heart's motion; and in this respect they might also be divided into two classes. Some of them act upon the heart before affecting the voluntary muscles; such is the case with digitalis and upas antiar. Corroval and vao enjoy this power in a still greater degree. The reverse is the case with other poisons; they act upon the voluntary muscles at first, and do not paralyze the heart till a latter period of the destructive process; the American arrows belong to this latter class. It is therefore easy to conceive how wide is the difierence between the intensity with which these poisons act in animals differently organized. A very small dose of corroval produces instant death in birds; a mammal survives a few minutes; while frogs resist the action of this substance for a considerable space of time, these animals, as you are aware, being able to survive a few hours after the total ablation of the heart.

We shall now perform a few experiments on various animals with these different poisons, and you will find that far from resembling woorara and strychnia, which destroy life without leaving the slightest vestige of their action, these muscular poisons actually produce deep physical changes in the tissue which loses its physiological properties under their influence.

(At this stage of the lecture a couple of pigeons were brought forward; an incision was made into the breast of both, laying bare the muscular fibres, and on applying test-paper, a decided alkaline reaction was met with in both cases. One of the pigeons was then killed with a poisoned arrow, the other with a solution of woorara injected into the cellular tissue. A frog was at the same time poisoned by introducing a small dose of corroval under the skin; it was then replaced in the jar from which it had been taken.)

You perceive, gentlemen, that the muscular tissue offers a decided akaline reaction in the healthy state. But in animals poisoned with any of those substances which act upon the contractile elements, the reaction of this tissue becomes acid, and the *rigor mortis* occurs immediately after death; two changes which spontaneously take place in dead animals, but only after a space of twenty-four hours has elapsed. The electrical properties of this tissue equally undergo a singular alteration, for, in the ordinary state of things, the external surface of a muscle is positively, and its internal or cut surface negatively electrified; the reverse is the case in animals poisoned with these toxic agents. And, lastly, on opening the bodies immediately after death, the heart is found contracted, motionless, rigid, and totally empty, so that its transparent walls in the frog have lost the ruddy color imparted by the presence of blood within the cardiac cavities, and appear perfectly white and colorless.

In all such cases the muscular element alone has been acted upon; for if on poisoning an animal with one of these agents, you were to apply a ligature round one of the limbs, and thus prevent the poison from reaching it, you would find that while the other muscles of the body remained insensible to the action of galvanism, those of the limb of thus preserved, would really obey its influence, when excited through the corresponding nerves; a proof that the muscular fibres alone have in this case been interfered with, the nerves retaining as before all their vital properties.

(The bodies of the poisoned animals were now opened; in the pigeon killed with an arrow, the heart was found rigid and motionless, the muscles stiff, and incapable of responding to the galvanic current. The same phenomena were observed in the frog, the heart of which was particularly rigid and perfectly colorless. On testing the muscles, their reaction was found decidedly acid. In the pigeon killed with woorara, the heart was found still in motion; and when at length its pulsation had ceased, galvanism was applied to it, and caused the parietes to contract; the muscles of the limbs responded readily and powerfully to the same agent and their reaction was alkaline.)—(*Medical Times and Gazette*, September 29, 1860.)—*N. A. Review.*

